# Optimal GHZ Paradox for Three Qubits

**DOI:** 10.1038/srep13080

**Published:** 2015-08-14

**Authors:** Changliang Ren, Hong-Yi Su, Zhen-Peng Xu, Chunfeng Wu, Jing-Ling Chen

**Affiliations:** 1 Center of Quantum information, Chongqing Institute of Green and Intelligent Technology, Chinese Academy of Sciences, People’s Republic of China; 2 Theoretical Physics Division, Chern Institute of Mathematics, Nankai University, Tianjin 300071, People’s Republic of China; 3 Pillar of Engineering Product Development, Singapore University of Technology and Design, 8 Somapah Road, Singapore 487372; 4 Centre for Quantum Technologies, National University of Singapore, 3 Science Drive 2, Singapore 117543.

## Abstract

Quatum nonlocality as a valuable resource is of vital importance in quantum information processing. The characterization of the resource has been extensively investigated mainly for pure states, while relatively less is know for mixed states. Here we prove the existence of the optimal GHZ paradox by using a novel and simple method to extract an optimal state that can saturate the tradeoff relation between quantum nonlocality and the state purity. In this paradox, the logical inequality which is formulated by the GHZ-typed event probabilities can be violated maximally by the optimal state for any fixed amount of purity (or mixedness). Moreover, the optimal state can be described as a standard GHZ state suffering flipped color noise. The maximal amount of noise that the optimal state can resist is 50%. We suggest our result to be a step toward deeper understanding of the role played by the AVN proof of quantum nonlocality as a useful physical resource.

The violation of Bell’s inequality exhibits a conflict between local realism and quantum theory^1^,^2^. However, such a conflict has only been displayed *statistically*. By devising effective logical arguments, an even sharper conflict between any local hidden variable model and quantum mechanical predictions can also be exhibited without resorting to inequalities. The most relevant examples of this line are Greenberger-Horne-Zeilinger (GHZ)^3^,^4^ and Hardy’s proofs^5^ of Bell nonlocality. These proofs are also referred to as the “all-versus-nothing” (AVN) proof^6^.

The AVN proof of, but not limited to, Bell nonlocality has attracted much attention and extensive results have been achieved both theoretically and experimentally. For instance, Cabello presented an AVN proof for two observers which holds for maximally entangled states^7^,^8^; Scarani *et al.* pointed out that any cluster state can display its nonlocality in the sense of GHZ paradox^9^; Cabello and Moreno presented the AVN proofs with *n* qubits distributed between *m* parties^10^. On the other hand, the experimental tests of AVN proofs have been demonstrated by the two-photon hyperentanglement^11^,^12^,^13^ and by energy-time entanglement^14^. As shown in the literatures, the AVN proof not only opened “a new chapter on the hidden variables problem” and made “the strongest case against local realism since Bell’s work”, but also played an active role in quantum information science, such as quantum protocols to reduce communication complexity^15^ and quantum key distribution protocols^16^. Furthermore, the AVN proof has been shown to be effective as well in the studies of multipartite entanglement^17^, quantum steering^18^,^19^,^20^ and quantum contextuality^21^.

However, most of the known results on the AVN proof are ideally based on pure states. In practical experiments, interaction between system and environment is unavoidable and hence pure entangled states inevitably become mixed states because of the effect of decoherence. So it is of significance to explore the AVN proof for mixed states. Although researchers’ understanding of pure states has been meaningfully improved in recent years, mixed states has remained a notoriously difficult subject. Impressively, Ghirardi and Marinatto considered a nonlocality test without inequality in the case of mixed states^22^. One year later, the AVN proof for multipartite mixed states have also been discussed^23^,^24^,^25^.

In this work, we present the optimal three-qubit GHZ paradox in the sense that an optimal mixed state can be found such that the logical inequality, formulated by the GHZ-typed event probabilities in Ref. 22, can be maximally violated for any fixed state mixedness.

## Results

### The optimal GHZ paradox

To investigate the *optimal* GHZ paradox of a three-qubit system, we start with the original case for the standard GHZ state


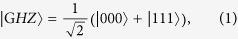


where |0〉 and |1〉 are the eigenstates of the Pauli matrix *σ*_*z*_ associated to the eigenvalues +1 and −1, respectively. Consider a set of four mutually commutative observables 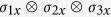, 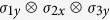, 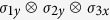, and 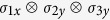, where *σ*_1*x*_ is defined as the Pauli matrix *σ*_*x*_ measured on the 1-st qubit (similarly for the others), and state (1) is the common eigenstate of these four operators, with the eigenvalues being +1, −1, −1, −1, respectively.

However, as shown in Ref. 4, a contradiction arises if one tries to interpret the quantum result with local hidden variable (LHV) models, in which each local observable has two definite values, +1 and −1, even before the measurements. Specifically, we denote the supposedly definite values of *σ*_1*x*_, *σ*_2*y*_, ··· as *v*_1*x*_, *v*_2*y*_, ···, then a product of the last three observables, according to LHV models, yields 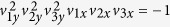, in sharp contradiction to the first observable *v*_1*x*_*v*_2*x*_*v*_3*x*_ = +1.

From the point of view of experiments, environment-induced noise is generally unavoidable, and the efficiency of generating three-qubit entangled states in the laboratory is usually below 90%. Hence it is important and highly nontrivial to take account of the case of mixed states when studying the GHZ paradox. Ghirardi and Marinatto^23^ demonstrated that the GHZ proof of nonlocality exists for a mixed state *ρ* if the following inequality


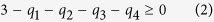


is violated, where *q*_*i*_ is defined as the event probability for each observable mentioned above that happens with certainty:


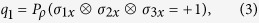



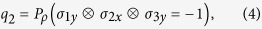



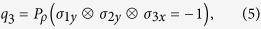



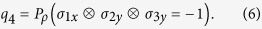


The degree of violation of (2) exhibits the degree of nonlocality.

Thus one may ask: What is the optimal state that violates (2) maximally so as to show the largest degree of nonlocality? To address the problem we use the notion of linear entropy^26^, which is a measure of state mixedness and computed as


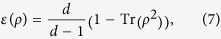


where the fraction is for normalization, with *d* = 2^*N*^ and *N* being the number of qubits. Put more precisely, the problem now becomes: Which state, for a fixed value of its linear entropy, can achieve the maximal violation of (2)? We usually need to solve a optimization problem by evaluating all states in the Hilbert space. The computation complexity increases very rapidly as the dimension of Hilbert space increases. Here we focus on three qubits and present a simple and rigorous method to obtain the optimal state, which reads


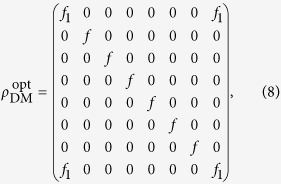


where 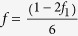, and subscript *DM* indicates that it is the form of physical density matrix giving maximal entropy for a given amount of violation.

The rest of the paper will be contributed to a proof of (8). But before the proof, we would like to give some discussions on the physical aspect of the state. Let us rewrite the optimal state (8) as


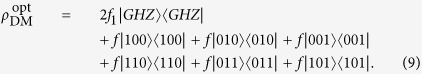


Its linear entropy, according to Eq. (7), equals


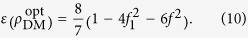


It is clear that the optimal state (9) is a mixture of the standard GHZ state and flipped noise equally upon six of eight bases.

Given the condition Eq. (17) (see below), we must have 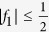. Hence our GHZ proof is valid only when 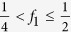. When 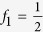, the optimal state is reduced to the standard GHZ state; when 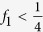, which corresponds to the case where the amount of flip noise weighs over 50%, the state could be compatible with a LHV model. Thus 50% is the upper bound of the amount of flipped noise that can be resisted by the optimal state.

### Proof of the optimal state

A three-qubit state can be represented as^27^,^28^


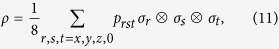


where *p*_*rst*_’s are real coefficients, *σ*_0_ denotes the identity matrix, and *p*_000_ = 1 for consistency. Of course, *p*_*rst*_ should be subject to some constraints: (i) *ρ* should be positive semi-definite, and (ii) the trace of *ρ* should be unity.

The linear entropy of state (11) equals the sum of square of each coefficient, i.e.,


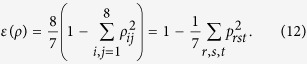


It is evident that the state that gives the maximal violation of (2) for a fixed linear entropy is equivalent to the state that gives the maximal linear entropy for a fixed quantum violation.

For the given state of the form (11), the four event probabilities defined by Eq. (3, 4, 5, 6) can be obtained as


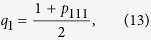



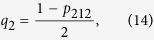



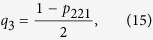



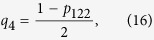


which imply that


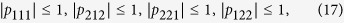


and substituting (13–16) into (2), we obtain


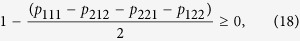


which implies that the violation only depends on four coefficients *p*_111_, *p*_212_, *p*_221_, *p*_122_. Only when *p*_111_ − *p*_212_ − *p*_221_ − *p*_122_ > 2 can inequality (18) be violated. Note that the algebraic maximum of *p*_111_ − *p*_212_ − *p*_221_ − *p*_122_ is 4. Intriguingly, the real part of matrix elements *ρ*_18_ and *ρ*_81_ can be expressed by 
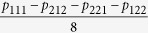
. For simplicity, we hereafter denote *p*_111_ − *p*_212_ − *p*_221_ − *p*_122_ by 8*f*_1_, and then inequality (18) becomes


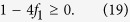


According to Eq. (12), for a fixed value of *f*_1_, matrix elements that do not contribute to *f*_1_ should be set to zero in order to maximize the linear entropy, i.e., the less the irrelevant matrix elements there are, the higher the linear entropy becomes. Therefore the following form of matrix seems to be the best solution: (see **Methods**)


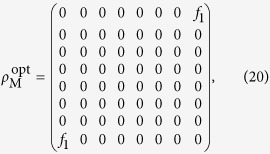


except the fact that it is not a density matrix.

As we know, a real density matrix is positive semi-definite and its trace is unity. According to the sufficient and necessary condition of positive semi-definite matrix and similar discussion above, it is easily and rigorously to obtain the form of optimal positive semi-definite matrix that has the maximal entropy for a given amount of violation *f*_1_, which can be expressed as


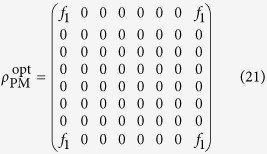


where one has to set *ρ*_11_ = *ρ*_88_ = *f*_1_. Compared with positive semi-definite matrix, density matrix has one more restrict, that is 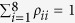. Obviously, only when *f*_1_ = 1/2, Eq. (21) represents a real physical density matrix, which means the optimal density matrix can reach that of positive semi-definite matrix Eq. (21). In this case it is nothing but the maximally entangled pure state which given the maximal violation of Eq. (18). However, when *f*_1_ < 1/2, the optimal positive semi-definite matrix is not a density matrix. To obtain the form of the optimal density matrix, the diagonal matrix elements should have nonzero terms and the sum of them must be 1, hence, in computing the linear entropy we note that


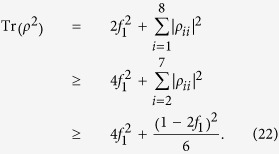


That is, due to the geometric-mean inequality, the maximal linear entropy can be obtained when *ρ*_11_ = *ρ*_88_ = *f*_1_ and *ρ*_22_, *ρ*_33_, ··· ,*ρ*_77_ are equal to each other, as is precisely the case of (8). Hence state (8) is indeed the optimal state that has the largest degree of nonlocality.

## Discussion

In conclusion, we have proved the optimal GHZ paradox by finding the hull of quantum states that saturate the trade-off relation between the linear entropy and the quantum violation of (2). The optimal state can be considered as the standard GHZ state suffering flipped color noise, and we have shown that the exhibition of the GHZ paradox for the optimal state depends on the amount of the noise: the stronger the noise is, the less nonlocality the optimal state has. When the amount of noise is over 50%, the optimal state does not bear the nonlocality that violates inequality (2). The method we use in the present paper provides a particularly new perspective to understand the GHZ paradox for mixed states, and our results may have potential applications in quantum information processing.

## Methods

### Derivation of 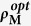


As we know, all matrix elements *ρ*_*ij*_ in Eq. (11) are the combinations of *p*_*rst*_’s.Because *ρ*_18_ and *ρ*_81_ depend on the four coefficients *p*_111_, *p*_212_, *p*_221_, *p*_122_, we only need to consider the matrix elements containing coefficients *p*_111_, *p*_212_, *p*_221_, *p*_122_, and set the others zero. Obviously, except the anti-diagonal matrix elements, the remaining matrix elements do not depend on such coefficients, so they can be set to zero.

Then we analyze whether the anti-diagonal matrix elements, except *ρ*_18_ and *ρ*_81_, can be zero. We solve the following equations


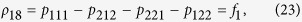



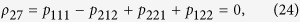



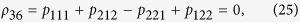



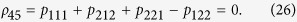


The solutions are found to be 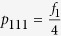 and 
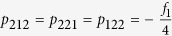
. Hence the matrix (20) is indeed the form of matrix that has the maximal linear entropy for a fixed amount of violation *f*_1_.

## Additional Information

**How to cite this article**: Ren, C. *et al.* Optimal GHZ Paradox for Three Qubits. *Sci. Rep.* **5**, 13080; doi: 10.1038/srep13080 (2015).
